# SIRT1 and Klotho expression in the heart and kidneys of rats with acute and chronic renovascular hypertension

**DOI:** 10.3325/cmj.2021.62.504

**Published:** 2021-10

**Authors:** Mahboobeh Yeganeh-Hajahmadi, Hamid Najafipour, Farzaneh Rostamzadeh, Yaser Masoumi-Ardakani

**Affiliations:** 1 Physiology Research Center, Institute of Neuropharmacology, Kerman University of Medical Sciences, Kerman, Iran; 2 Cardiovascular Research Center, Institute of Basic and Clinical Physiology Sciences, Kerman University of Medical Sciences, Kerman, Iran; 3 Endocrinology and Metabolism Research Center, Kerman University of Medical Sciences, Kerman, Iran; 4 Physiology Research Center, Institute of Neuropharmacology, Kerman University of Medical Sciences, Kerman, Iran

## Abstract

**Aim:**

To evaluate Klotho and SIRT1 expression in the heart and kidneys of rats with acute and chronic renovascular hypertension.

**Methods:**

Four and sixteen weeks after the induction of renovascular hypertension by clipping the left renal artery, systemic blood pressure, serum angiotensin II level, and the expression of Klotho and SIRT1 proteins and oxidative stress indices in the heart and kidneys were assessed.

**Results:**

SIRT1 level was significantly reduced in the ischemic (left) kidney in acute and chronic phases of hypertension. In the heart, it decreased in the acute phase, but increased in the chronic phase. Klotho levels in the heart and kidneys did not change significantly in either hypertension phase. Superoxide dismutase (SOD) activity in the heart significantly decreased, and SOD, total antioxidant capacity, and malondialdehyde in the ischemic kidney significantly increased during the development of hypertension. Serum angiotensin II level significantly increased in the acute phase of hypertension.

**Conclusion:**

Development of renovascular hypertension was associated with a reduction of SIRT1 expression in the heart and ischemic kidney. As angiotensin II and SIRT1 counteract each other's expression, a SIRT1 reduction in the heart and kidney, along with the influence of systemic/local angiotensin II, seems to be partly responsible for hypertension development. A combination of SIRT1 agonists and angiotensin II antagonists may be considered for use in the treatment of renovascular hypertension.

Hypertension is one of the leading causes of disease burden worldwide, doubling the risk of coronary artery diseases (1). The prevalence of hypertension in US adults in the 2013-2016 period ranged from 26.1% in the age group 20-44 to 78.2% among people older than 65 years (2). Despite antihypertensive treatment, blood pressure of more than half of American adults is not controlled (3). Thus, to be able to produce more effective drugs, the underlying mechanisms of hypertension should be investigated.

The most common cause of death in hypertensive patients is hypertensive heart disease, which results from functional and structural adaptation of the heart to high blood pressure (1). Secondary hypertension is most frequently a result of primary kidney disease. On the other hand, hypertension is a risk factor for kidney damage and end-stage renal disease (1).

Hypertension and related cardiovascular diseases are age-dependent (4,5). The aging of the cardiovascular system is an important process determining longevity (6).

Sirtuins are a family of enzymes encoded by SIRT1 to SIRT7 in mammals that play important roles in longevity (7). These enzymes are abundantly expressed in the nucleus and cytoplasm of several tissues, including the heart and vascular endothelium (8). The most well-known member of the sirtuin family is SIRT1, which plays beneficial roles in age-associated metabolic, inflammatory, and cardiovascular diseases (9). SIRT1 has anti-oxidant, anti-inflammatory, and anti-apoptotic effects in the endothelium and prevents endothelial senescence and dysfunction (10,11). Several studies showed that SIRT1 protected against atherosclerosis (10-13). Increasing SIRT1 expression in mice improved vascular remodeling and hypertension caused by angiotensin II (14). In addition, through reducing SIRT1 expression, hyperglycemia causes vascular damage (15).

Klotho is a membrane-bound protein that exerts anti-aging function (16). Klotho deficiency leads to premature aging phenotype and shortens the lifespan (17), while its increased gene expression increases life expectancy (18). Klotho is involved in the prevention of arteriosclerosis, inducing its effects even in tissues that do not express it, which indicates its endocrine role (16). A recent study on Klotho haplodeficient mice showed that Klotho deficiency led to arteriosclerosis and hypertension, but these effects were diminished by increasing SIRT1 activity (19).

One of the experimental models to evaluate secondary hypertension is 2-kidney-1-clip (2K1C) hypertension (20). In this model, a clamp is placed on one of the renal arteries to induce ischemia, while the other renal artery remains intact. This procedure steadily increases blood pressure due to an increased activity of the renin-angiotensin system in the acute phase, and sodium and water retention in the chronic phase (20,21). As SIRT1 and Klotho play a role in blood pressure regulation, and the kidneys play a role in secondary hypertension, we hypothesized that these two proteins may partake in the development of acute and chronic renovascular hypertension. Therefore, the aim of this study was to assess the expression of these two proteins in the heart and in the ischemic and non-ischemic kidneys of 2K1C rats. On the other hand, it has been shown that angiotensin II infusion increases oxidative stress and blood pressure, and that the deleterious effects of angiotensin II on blood pressure and the kidneys can be prevented by an inhibition of reactive oxygen species after angiotensin II infusion (22) and in 2K1C rats (23). Furthermore, it has been shown that SIRT1 exerts its beneficial effects by reducing oxidative stress (11,24). Therefore, the amount of oxidative stress in the heart and kidneys of the experimental animals was also assessed.

## Material and methods

Male Wistar rats (180-200 g) were purchased from Kerman Physiology Research Center. The animals were housed under a 12-h light/dark cycle, with standard rat chow (containing normal sodium) and water *ad libitum*. All experiments were performed according to the national guidelines for animal studies. The study was approved by the Ethics Committee of the Kerman University of Medical Sciences (IR.KMU.REC.1397.039). Twenty-eight rats were randomly allocated into four groups of seven animals: 1) acute sham, 2) acute hypertension, 3) chronic sham, and 4) chronic hypertension.

### Induction of hypertension

Hypertension was induced by applying a plexiglass clip on the left renal artery (25). Briefly, the animals were anesthetized with an intra-peritoneal injection of ketamine (80 mg/kg) and xylazine (10 mg/kg). The abdominal wall of the flank was incised, and a clip with a 0.2-mm cleft diameter was placed around the left renal artery to induce partial ischemia. The sham groups were subjected to the same procedure, without clip placement. The induction of acute and chronic renovascular hypertension lasted four and sixteen weeks, respectively (26). At the end of the forth or 16th week, the animals were anesthetized with an intra-peritoneal injection of sodium thiopental (50 mg/kg). A polyethylene catheter (PE-50) was placed in the right femoral artery and connected to a pressure transducer to record arterial blood pressure for 20 minutes. Only rats with a systolic arterial pressure >150 mm Hg were included in the hypertension groups, as some animals may not develop hypertension due to too loose (no ischemia) or too severe (renal necrosis) renal artery occlusion. After blood pressure was recorded, the animals were sacrificed under deep anesthesia. The heart and kidneys were removed, washed in saline, and wiped using a sterilized and soft cloth. The atria and right ventricle of the heart were removed using small surgical scissors. The left ventricle + septum and both kidneys were weighed and quickly immersed in liquid nitrogen and stored at -80 °C for molecular experiments. The left-to-right kidney weight ratio was used as an index of kidney ischemia. The ratio of left ventricle + septum weight to body weight was used as an index of left ventricular hypertrophy.

### Western blotting for Klotho and SIRT1 expression assessment

Tissue samples were homogenized in ice-cold RIPA lysis buffer containing a protease inhibitor. The homogenates were centrifuged at 14 000 × g for 20 min at 4 °C. The lysate protein concentration was determined using the Bradford method (Bio-Rod Laboratories, Munich, Germany). An equal volume of 2X SDS sample buffer was added, and then the mixture was boiled for 5 min. The samples were resolved electrophoretically on a 12.5% SDS-PAGE gel and transferred to PVDF membranes. The membranes were blocked overnight at 4 °C with 5% nonfat powdered milk in TBST and then incubated for 3 h with a primary antibody (SIRT1 or Klotho, 1:500; Santa Cruz Biotechnology Inc., Dallas, TX, USA) at room temperature. After washing in TBST for three times (10 min each), the blots were incubated with goat anti-mouse IgG secondary antibody (1:10000; Santa Cruz Biotechnology Inc.) for 1 h at room temperature. All antibodies were diluted with blocking buffer. The antibody-antigen complex was detected using Western blot documentation system and analyzed with imageJ analyzing software. β-actin immunoblotting was used as a loading control (27). Serum concentration of angiotensin II, was determined with the ELISA kit (Ray Biotech, Inc., Norcross, GA, USA)

### Oxidative stress evaluation

Superoxide dismutase (SOD) activity (as an antioxidant) was measured using a colorimetric assay kit (Teb Pazhouhan Razi, Tehran, Iran). In brief, 50 mg of heart or kidney tissue was homogenized in 250 μL lysis buffer on ice. Thereafter, the samples were centrifuged at 14 000 × g for 5 min at 4 °C. SOD activity was measured using 10 μL of the supernatant according to the manufacturer’s instructions.

Total antioxidant capacity (TAC) of the tissues was measured by an appropriate commercial kit (Teb Pazhouhan Razi). In brief, 100 mg of the tissues was homogenized in 500 μL ice-cold 1X assay buffer and centrifuged at 12 000 × g for 15 min at 4 °C. TAC was measured at 415 nm using 10 μL of supernatant, according to the manufacturer’s instructions.

To estimate malondialdehyde (MDA, Teb Pazhouhan Razi) level (as an oxidant), after homogenizing the tissues and centrifuging, 100 μL of the supernatants was used. The procedure was performed according to the manufacturer’s instructions, and the concentration of MDA-thiobarbituric acid was measured at 530 nm.

### Statistical analysis

The normality of distribution was tested with the Kolmogorov-Smirnov test. The values are expressed as mean ± standard deviation. The unpaired *t* test was used for comparison of two groups, and one-way ANOVA test followed by Tukey’s *post-hoc* analysis for comparison among the studied groups. All *P* values were two-tailed, and *P* < 0.05 was considered as the significance level. The statistical analysis was performed with SPSS, version 20.0 (IBM Corp., Armonk, NY, USA).

## Results

### Basic parameters

Clipping the left renal artery significantly increased systolic and diastolic blood pressure in rats with acute and chronic 2K1C compared with their corresponding sham groups. Clipping also decreased the weight of the left (ischemic) kidney compared with the right (normal) kidney, and decreased the left-to-right kidney weight ratio and left kidney weight-to-body weight ratio (Table 1).

**Table 1:** The effect of clipping the left renal artery on systolic and diastolic blood pressure, left kidney-to-right kidney weight ratio, and left ventricle hypertrophy index in the hypertension and sham groups

Variables	Sham acute	Hypertension acute	Sham chronic	Hypertension chronic
LKW/RKW (mg/mg)	0.94 ± 0.16	0.62 ± 0.58^†^	1.04 ± 0.08	0.61 ± 0.21^†^
LKW/BW (mg/g)	3.64 ± 0.29	2.70 ± 0.82^†^	3.26 ± 0.21	2.37 ± 0.26^†^
RKW/BW (mg/g)	3.88 ± 0.26	4.50 ± 0.79	3.4 ± 0.08	3.9 ± 0.52^†^
LVW/BW (mg/g)	2.35 ± 0.45	2.47 ± 0.61	2.05 ± 0.08	2.35 ± 0.29^†^
Systolic blood pressure (mmHg)	114.6 ± 21.9	169.5 ± 12.4^†^	110.8 ± 8.2	171.2 ± 14.0^†^
Diastolic blood pressure (mmHg)	80.3 ± 16.4	121.7 ± 10.8^†^	75.3 ± 5.8	120.9 ± 12.7^†^

*Abbreviations: LKW – left kidney weight; RKW – right kidney weight; LVW – left ventricle weight; BW – body weight, LW – lung weight.

†*P* < 0.01 vs sham.

### Klotho and SIRT1 levels in the heart and kidneys

**Figure 1:**
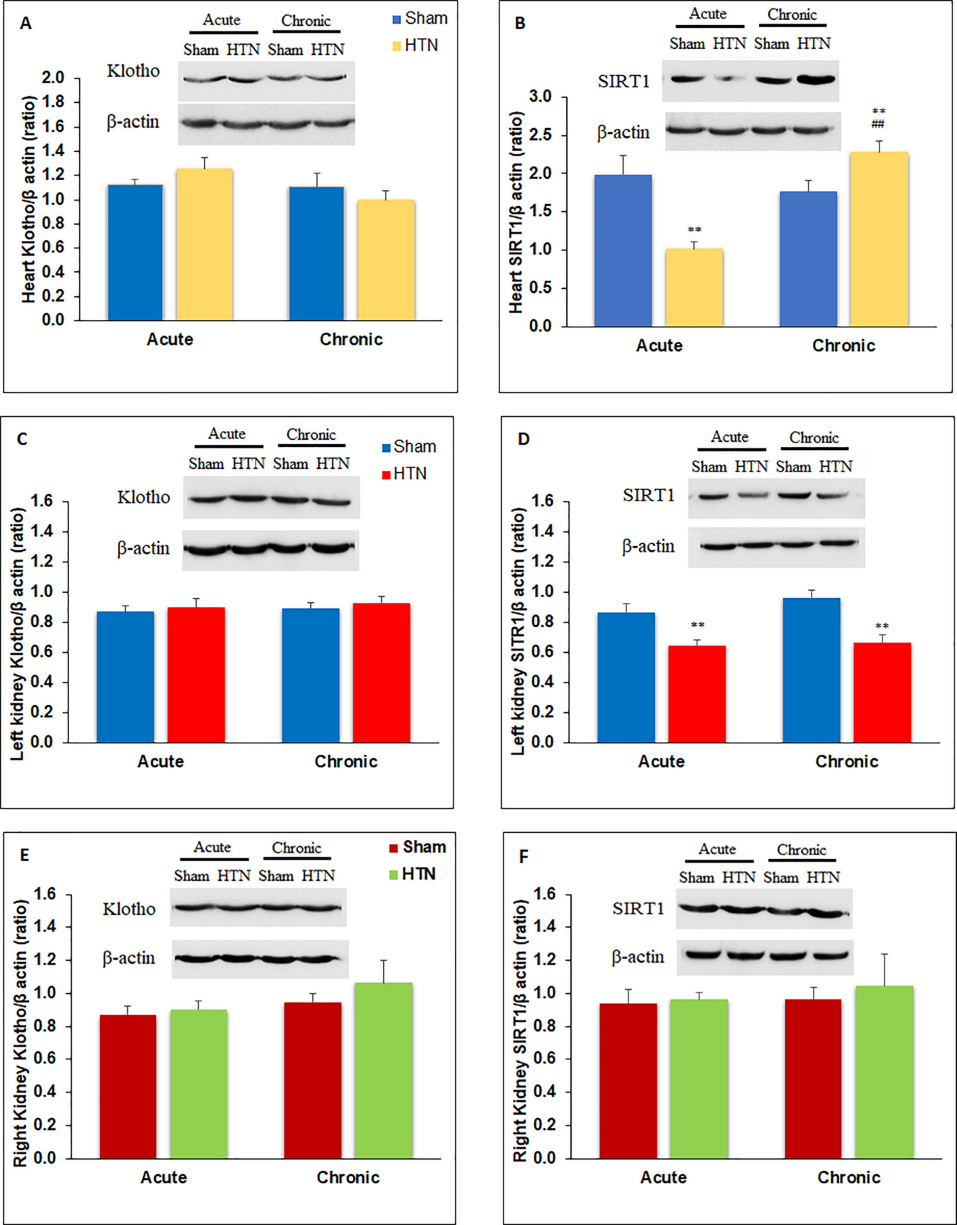
Klotho (A, C, E) and SIRT1 (B, D, F) expression in the heart (up), left kidney (middle), and right kidney (down) in rats with hypertension (HTN) and the sham group (n = 7). **P < 0.01 vs corresponding sham, ## *P* < 0.01 vs acute hypertension.

Klotho expression in the heart in hypertension groups was not different from that in the corresponding sham groups (Figure 1A). SIRT1 expression in the heart of rats with acute hypertension was significantly lower and SIRT1 expression in the heart of rats with chronic hypertension was significantly higher than in their corresponding sham groups (Figure 1B).

Klotho expression in the ischemic (left) and non-ischemic (right) kidney of rats with acute hypertension was not different from that in their corresponding sham groups. SIRT1 level in the kidney of non-ischemic groups also did not change. However, SIRT1 level was significantly reduced in the ischemic kidneys in both acute and chronic conditions (Figure 1D).

### Oxidative stress levels in the heart and kidneys

**Figure 2:**
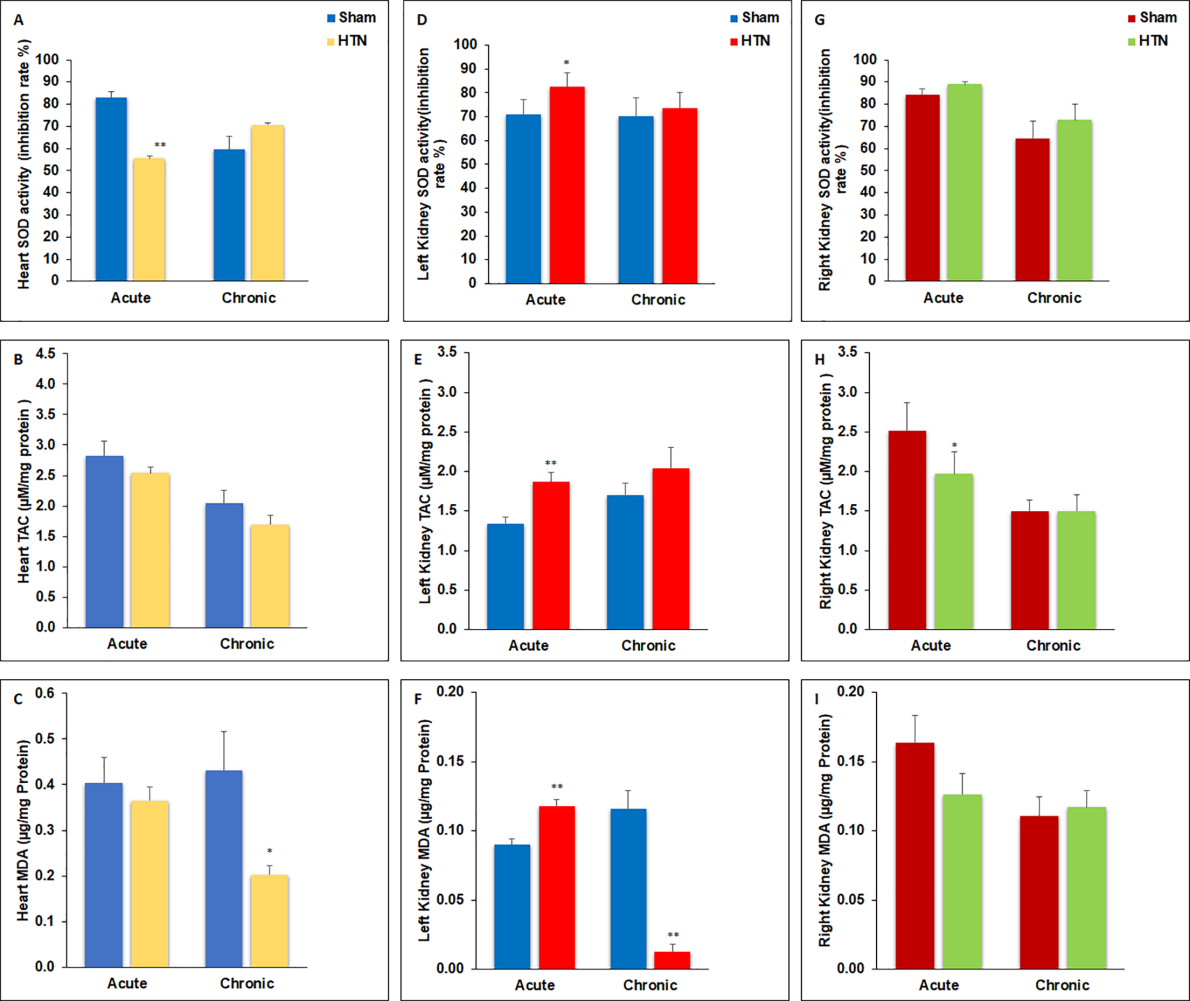
Antioxidant and oxidant status of the heart (A-C), left kidney (ischemic, D-F), and right kidney (non-ischemic, G-I) in rats with hypertension (HTN) and the sham group. SOD – superoxide dismutase activity, TAC – total antioxidant capacity, MDA – malondialdehyde levels. (n = 7); *P < 0.05 vs sham and **P < 0.01 vs sham.

SOD activity in the heart of rats with acute hypertension (Figure 2A) and MDA levels in the heart of rats with chronic hypertension (Figure 2C) were significantly reduced. TAC did not significantly change in the heart of either acute or chronic hypertension rats (Figure 2B). SOD activity (Figure 2D), TAC (Figure 2E), and MDA levels (Figure 2F) significantly increased in the ischemic kidney of rats with acute hypertension, and MDA levels significantly decreased in the ischemic kidney (Figure 2F) of rats with chronic hypertension. Total antioxidant activity was reduced significantly in the right kidney of rats with acute hypertension (Figure 2H).

### Serum angiotensin II

**Figure 3:**
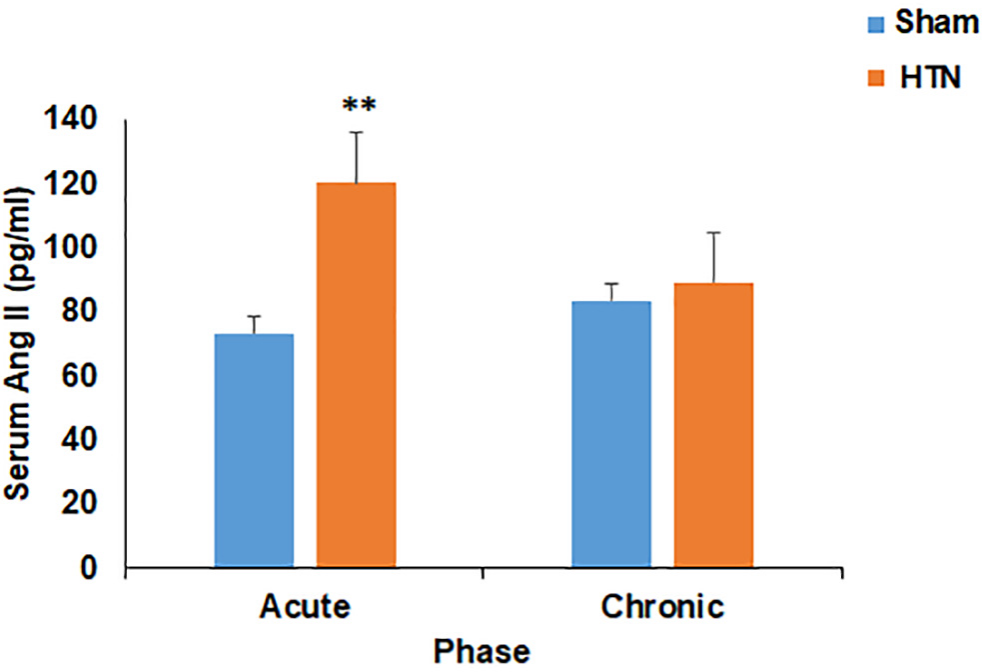
The level of angiotensin II in the serum of rats with hypertension (HTN) and the sham group four (acute) and 16 weeks (chronic) after left renal artery clipping (n = 6 in each group); **P < 0.05 vs sham.

In the acute phase of hypertension, angiotensin II level significantly increased in the hypertension group compared with the sham group. However in the chronic phase, it did not significantly differ between the groups, ie, angiotensin II level returned to an almost normal level (Figure 3).

## Discussion

In this study, Klotho levels in the heart and kidneys of acute and chronic hypertension groups did not significantly differ from those in their corresponding sham groups. SIRT1 expression in the heart decreased in the acute phase and increased in the chronic phase of renovascular hypertension. SIRT1 expression in the ischemic kidneys decreased in both acute and chronic phases, but in the non-ischemic kidneys it did not significantly change either in the acute or the chronic phase.

Studies report on different SIRT1 levels in various pathologic conditions. For example, these levels are upregulated in diabetes-induced cardiac remodeling (28) and in cancers (29,30). The current study showed reduced SIRT1 levels in the heart in acute hypertension and increased SIRT1 levels in chronic hypertension. Unilateral renal artery constriction initially increases blood pressure in response to the activation of renin-angiotensin system (26,27). A key factor in the induction of acute renovascular hypertension is angiotensin II. However, in chronic renovascular hypertension, which occurs nine weeks or more after renal artery constriction, high blood pressure results from sodium and water retention and increase in plasma volume. In this stage of hypertension, serum angiotensin II levels return to normal, as was shown by other investigators (26,31), but local angiotensin II in the kidneys remains high (20,21). It has been reported that angiotensin II reduces SIRT1 expression in the aorta (14), and these regulatory factors counteract each other’s expression. In acute renovascular hypertension, high angiotensin II levels in blood and tissues decrease SIRT1 expression in the heart and kidneys. In chronic hypertension, high blood pressure and high volume loads lead to cardiac hypertrophy. These processes were in the present study accompanied by an increase in SIRT1 levels in the heart. Consistent with these findings, it has been shown that SIRT1 expression increases in the heart of spontaneously hypertensive rats (a chronic condition), and that left ventricular hypertrophy positively correlates with SIRT1 expression (32). It has also been reported that in humans, SIRT1 levels increase in (chronic) hypertension, which is associated with cardiac hypertrophy (33).

SIRT1 expression in the non-ischemic kidney of rats did not change in either acute or chronic condition. However, in ischemic kidney it was decreased in both hypertension phases, and in the heart it was decreased in the acute and increased in the chronic phase. In conclusion, changes in the heart SIRT1 respond to blood angiotensin II levels and are not directly related to what happens in the ischemic kidney. Considering the high activity level of local renin-angiotensin system in the clipped kidney (34), it is possible that local angiotensin II governs SIRT1 expression in the ischemic kidney by a counter-regulatory effect.

In this study, Klotho levels in the heart and kidneys did not change significantly in acute and chronic hypertensive conditions. This is in agreement with the findings of Aizawa et al (35), who showed that kidney Klotho levels in spontaneously hypertensive rats (SHR) were not different from those of normal Wild Kyoto rats at 18 weeks, but were lower after 60 weeks. Some studies have shown that Klotho levels in the kidneys decreased after ischemia reperfusion injury (IRI) (36,37). In IRI, blood supply to tissues is blocked for a short time (several minutes), but in 2K1C the renal artery is narrowed for a longer period (4 and 16 weeks) and the blood flow is partially reduced. In addition, in IRI reperfusion is established, which *per se* can activate some deleterious processes, while in 2K1C, the blood flow is not re-established to a normal level. Thus, it seems that the mechanisms underlying the effects of IRI and long partial reduction of blood supply on kidneys' Klotho level are different, and they need to be further investigated.

The current study found SOD activity to be significantly reduced in the heart of acutely hypertensive rats. TAC (non-enzymatic antioxidant biomarker) and MDA levels (lipid peroxidation biomarker) did not change. In chronic hypertension, heart SOD activity increased and MDA level decreased. SOD changes in the heart might be related to SIRT1 changes. It has been shown that increased SIRT1 activity in endothelial cells increases SOD expression (38). More experiments are needed to explore the temporal relationship between SIRT1 and oxidant status in the heart of 2K1C rats.

In the current study, SOD activity and TAC increased in the ischemic kidneys of the acute hypertension group. Considering the reduced SIRT1 in this circumstances, it seems that changes in the antioxidant system in the ischemic kidney, unlike those in the heart, are not under SIRT1 control. This difference can probably be explained by the existence of different SOD isoenzymes in the heart and kidneys (39). Furthermore, significant increase in MDA indicates that it is possible for increased oxidative stress to increase SOD activity and TAC as a compensatory mechanism (40). Different oxidant status in the left and right kidney can be explained by the left kidney being ischemic and the right kidney having normal blood flow and being indirectly influenced by systemic hypertension and renin production by the left kidney. In addition, some studies have shown differences in the biologic development and function of the left and right kidney (41,42).

MDA is commonly used as an indicator of oxygen free radical damage to cell membranes. In the present study, MDA level decreased in the hearts of animals with chronic hypertension, which was associated with a SIRT1 increase. Furthermore, MDA significantly increased in the ischemic kidney of animals with acute hypertension, which was accompanied by a SIRT1 decrease. These results are consistent with other studies, which have shown that increased SIRT1 expression is associated with decreased MDA in cardiac ischemia reperfusion injury and in lung epithelial cells exposed to paraquat pesticide (43). However, in the ischemic kidney of chronic hypertension group, a significant MDA decrease was associated with SIRT1 decrease, indicating other pathways involved in chronic conditions.

Finally, as it has been shown that clamp removal or angiotensin II inhibition can reverse the changes in blood pressure in 2K1C animals (26) and that angiotensin converting enzyme (ACE) inhibition can revert angiotensin II-induced SIRT1 downregulation (44), ACE inhibitors/angiotensin receptor blockers are likely able to restore SIRT1 levels in renovascular hypertension.

One of the limitations of this study was that we did not evaluate different isoforms of SOD in the heart and kidneys. This type of assessment could help us understand why the relationship between SIRT1 and antioxidant system is different in the heart and kidneys. Furthermore, we observed an association between SIRT1 and Klotho with antioxidant system activity. To find out the probable causative link, more detailed experiments are needed.

Overall, the results of this study showed that the development of renovascular hypertension was associated with a SIRT1 reduction in the heart and ischemic kidney. As angiotensin II increases in the acute phase of this type of hypertension, and angiotensin II and SIRT1 counteract the expression of each other, SIRT1 reduction in the heart and kidney along with the influence of angiotensin II may take part in the establishment of hypertension. A combination of SIRT1 agonists and angiotensin II antagonists may be considered for use in the treatment of hypertension, especially the renovascular type.
